# Representation of viruses in the remediated PDB archive

**DOI:** 10.1107/S0907444908017393

**Published:** 2008-07-17

**Authors:** Catherine L. Lawson, Shuchismita Dutta, John D. Westbrook, Kim Henrick, Helen M. Berman

**Affiliations:** a RCSB Protein Data Bank, Department of Chemistry and Chemical Biology, Rutgers, The State University of New Jersey, 610 Taylor Road, Piscataway, NJ 08854-8087, USA; b Macromolecular Structure Database–European Bioninformatics Institute, EMBL Outstation–Hinxton, Cambridge CB10 1SD, England

**Keywords:** virus structures, Protein Data Bank, database integration, uniform curation, point symmetry, helical symmetry, biological assemblies

## Abstract

A new scheme has been devised to represent viruses and other biological assemblies with regular noncrystallographic symmetry in the Protein Data Bank (PDB). The scheme describes existing and anticipated PDB entries of this type using generalized descriptions of deposited and experimental coordinate frames, symmetry and frame transformations. A simplified notation has been adopted to express the symmetry generation of assemblies from deposited coordinates and matrix operations describing the required point, helical or crystallographic symmetry. Complete correct information for building full assemblies, subassemblies and crystal asymmetric units of all virus entries is now available in the remediated PDB archive.

## Abstract 2

A new data model for PDB entries of viruses and other biological assemblies with regular noncrystallographic symmetry is described.

## 1. Introduction

Recent improvements in structural biology methods have given rise to an increasing body of structural data for bio­logical assemblies composed of tens to thousands of individual protein and/or nucleic acid polymer chains. Structures of such quaternary complexes or assemblies present many challenges for archival representation and validation, graphical display and analysis (Dutta & Berman, 2005 ▶).

Large biological assemblies are often composed of multiple copies of one or more polymer entities, with the arrangement of repeating units following a regular point or helical symmetry (Goodsell & Olson, 2000 ▶). The largest class of biological assemblies with regular symmetry currently represented in the Protein Data Bank (PDB) archive (Berman *et al.*, 2000 ▶) are the icosahedral viruses, with approximately 250 structures determined either by X-ray crystallography or cryoelectron microscopy (CryoEM; reviewed by Harrison, 2001 ▶; Chiu & Rixon, 2002 ▶; Lee & Johnson, 2003 ▶). A smaller group of virus entries have helical symmetry: approximately 30 structures determined mainly by fiber X-ray diffraction methods (Marvin, 1998 ▶; Stubbs, 1999 ▶).

Other assemblies with regular noncrystallographic symmetry are also represented in the PDB. These include viral toxins with circular symmetry (Tilley *et al.*, 2005 ▶), clathrin cages and chaperonins with dihedral symmetry (Fotin *et al.*, 2006 ▶; Braig *et al.*, 1994 ▶), ferritins with tetrahedral or octahedral symmetry (Johnson *et al.*, 2005 ▶; Hamburger *et al.*, 2005 ▶) and DNA-processing enzymes with helical symmetry (Van Loock *et al.*, 2003 ▶; Conway *et al.*, 2004 ▶).

Assemblies may have multiple embedded symmetries or adjacent symmetries. For instance, the icosahedral *Paramecium bursaria* chorella virus type 1 (PBCV-1) algal virus shell has thousands of copies of a membrane-embedded coat protein arranged with pseudocrystalline symmetry (Nandhagopal *et al.*, 2002 ▶). The T4 tailed bacteriophage has fivefold, sixfold and helical symmetries aligned along a common axis (Leiman *et al.*, 2003 ▶).

The PDB entries of icosahedral and helical viruses and a handful of other large biological assemblies with regular noncrystallographic symmetry were previously archived in an inconsistent manner and were prone to errors. To address these problems, we have developed a flexible scheme to represent assemblies with regular symmetry. The scheme involves four key elements: (i) a set of atomic coordinates representing the repeating unit, (ii) parameters defining the regular symmetry, (iii) an operations list containing regular symmetry operations plus any frame transformations (transformations between different coordinate frames) and (iv) a compact set of assembly-generation instructions, with the possibility of defining multiple assemblies. Using this scheme, instructions may be given to build a full icosahedral virus in the deposited frame, a pentamer subassembly of the virus in the standard icosahedral point frame and the asymmetric unit of the virus crystal in the standard space-group frame.

This representation was developed to provide uniformity among virus structures within the PDB as part of a larger remediation project to remove legacy errors and improve the uniformity of the entire archive (Henrick *et al.*, 2008 ▶). The representation has been fully implemented in the PDB exchange dictionary and has been incorporated in the remediated entries of over 280 structures, mainly viruses but also several nonvirus assemblies (Table 1 ▶). The new scheme will permit routine annotation of future entries with regular and complex symmetries and will also make it possible to more easily build and view such assemblies within graphical display programs.

## 2. Background: remediation of virus entries

A review of 250 icosahedral virus structure entries and 30 helical virus entries deposited into the PDB between 1984 and 2006 revealed three major issues to be addressed in remediation: missing or erroneous sets of transformation operations, inconsistency in coordinate-frame representations and overly complex building instructions. For each issue, corrected information was gathered and validated in a systematic way.

For approximately 40% of virus entries, the set of matrix transformations needed to build up the full biological assembly either was absent or contained errors. Problem entries were identified by inspection of images generated *via* an automated script using the *Multiscale Model* module of *Chimera* (http://www.cgl.ucsf.edu/chimera/; Goddard *et al.*, 2005 ▶; Pettersen *et al.*, 2004 ▶). Corrected transformations were obtained from the Virus Particle Explorer database (VIPERdb; http://viperdb.scripps.edu; Reddy *et al.*, 2001 ▶; Natarajan *et al.*, 2005 ▶; Shepherd *et al.*, 2006 ▶) or the Protein Quaternary Structure server (PQS; http://pqs.ebi.ac.uk; Henrick & Thornton, 1998 ▶). For helical viruses, parameters to construct representative matrix transformations were collected from PQS.

**Figure 1:**
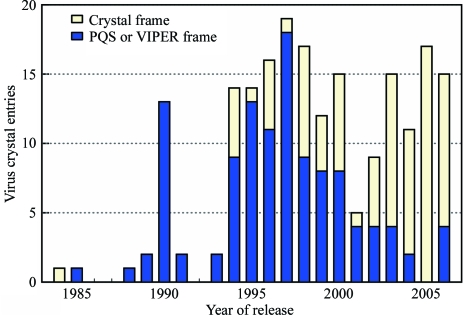
Deposition frame of remediated icosahedral virus crystal structure entries. The number of entries is plotted by year of release and coordinate frame type. Entries with coordinates provided in the standard frame of the crystal lattice are represented by light yellow bars. Entries presented in an icosahedral frame and requiring one or more non-identity transformations to place virus particles into the crystal lattice are represented by dark blue bars.

The atomic coordinates of virus entries have been archived in a variety of different coordinate reference frames. CryoEM structures and early crystal structures of icosahedral viruses are typically presented in one of two standard icosahedral reference frames. However, the recent trend for crystal structures is to deposit in the frame of the crystal lattice (Fig. 1 ▶). For each icosahedral virus, the transformation [*P*] that moves the deposited coordinates into the VIPER standard icosahedral frame was determined using the *PDB*2*VIPER* program (Shepherd *et al.*, 2006 ▶) with minor modifications. 60 transformations [*T*_*m*_], *m* = 1–60, were calculated for each assembly from a standard ordered set of icosahedral operations [*I*_*m*_] (see §[Sec sec3.1.1]3.1.1 for definition),
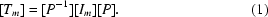
For 210 icosahedral virus crystal structures, transformations to the crystal lattice frame were collected from author text remarks or primary citations, extracted from SCALE records, or set to identity, as appropriate. One transformation was defined for each independent particle in a crystal asymmetric unit. Noncrystallographic symmetry (NCS) operations defining crystal asymmetric units were determined automatically using software developed in-house. Crystal packing was inspected using the *Crystal Contacts* module of *Chimera*. Of 88 crystal structure entries with deposited structure factors, 70 yielded *R* factors below 0.40 (56 below 0.30) using *SFCHECK* (Vaguine *et al.*, 1999 ▶). Before remediation, only a handful of these entries yielded reasonable validation statistics.

For the majority of virus-structure entries with atomic coordinates representing one regular symmetry (point or helical) asymmetric unit, application of regular symmetry operations is all that is required to build a full or representative assembly. However, several entries contain explicit atom coordinates for larger assemblies, *e.g.* an icosahedral pentamer, or a full crystal asymmetric unit with one quarter or one half of a full virus capsid. In some of these cases coordinates were presumably duplicated for convenient viewing of a particular interface, but in others regular symmetry is only approximate and explicit coordinates are required to represent the unique part of a lower symmetry structure. For the PBCV-1 virus (PDB code 1m4x; Nandhagopal *et al.*, 2002 ▶), atomic coordinates are only provided for a small fraction (1/28th) of one icosahedral asymmetric unit containing three chains: a total of 3 × 28 × 60 chains and 16 284 240 atoms are required to build the complete capsid. In all of these special situations, symmetry-parameter representation and instructions for building complete assemblies from selections of matrix operations, selections of coordinates and/or hierarchical application of transformation operations were defined on a case-by-case basis.

## 3. Representation of complexes with regular symmetry

In order to archive the corrected information gathered in the virus remediation process, the PDB exchange dictionary was extended (http://mmcif.pdb.org). New terms enable explicit definition of regular noncrystallographic point and helical symmetries and provide for definition of transformation operations and implementation of a compact notation for assembly generation. The new dictionary categories are used in conjunction with existing data items for crystal symmetry and logical groups of atomic coordinates. The resulting representation permits the description of biological assemblies with any regular symmetry and determined by any experimental method. An example of the representation in mmCIF format is provided as supplementary material.^1^

### 3.1. Regular symmetry definitions

Regular symmetries include point, helical and crystal symmetries. Given parameters appropriate to the symmetry type and a standard reference frame with a defined relationship between symmetry axes and Cartesian coordinate axes, a complete set of symmetry operations can be defined for any point group and representative symmetry operations can be defined for any helical or crystal symmetry. The PDB follows standard definitions for crystal symmetry (Hahn, 2002 ▶). Parameter and standard frame definitions used for point and helical symmetries are described below and follow the conventions for cryoEM structural studies proposed by Heymann *et al.* (2005 ▶).

#### 3.1.1. Point symmetry

**Figure 2:**
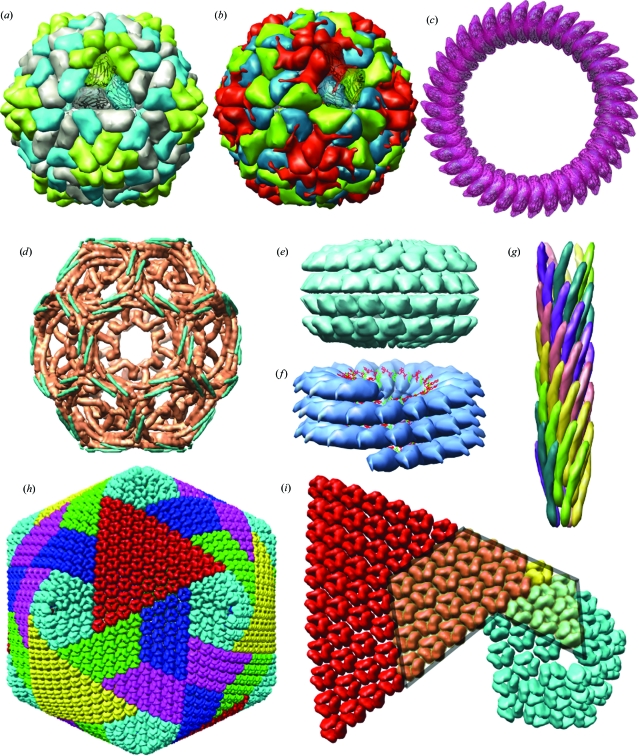
Examples of remediated PDB entries with regular noncrystallographic symmetry. (*a*) 1f2n, yellow mottle virus with icosahedral symmetry (Qu *et al.*, 2000 ▶). (*b*) 4rhv, rhinovirus with icosahedral symmetry (Arnold & Rossmann, 1988 ▶). In (*a*) and (*b*), the icosahedral asymmetric unit is shown in ribbon representation. (*c*) 2bk1, viral toxin pneumolysin with *C*38 circular symmetry (Tilley *et al.*, 2005 ▶). (*d*) 1f2n, clathrin cage with *D*6 symmetry (Fotin *et al.*, 2006 ▶). (*e*) 1ei7, tobacco mosaic virus coat protein four-layer aggregate with *D*17 symmetry (Bhyravbhatla *et al.*, 1998 ▶). (*f*) 1cgm, cucumber green mottle mosaic virus (CGMMV) with helical symmetry (Wang & Stubbs, 1994 ▶). Nucleic acid positions are shown in green and red. (*g*) 1ifd, filamentous phage with helical symmetry and fivefold circular symmetry (Marvin, 1990 ▶). Each color represents a strand winding about the helical axis. (*h*) 1m4x, *P. bursaria* chorella virus type 1 (PBCV-1) algal virus shell (Nandhagopal *et al.*, 2002 ▶). Colors highlight pentasymmetron units (cyan) and trisymmetron units (red, yellow or blue–green–magenta). (*i*) Adjacent PBCV-1 pentasymmetron and trisymmetron. The position of the deposited coordinates for the protein trimer is shown in yellow. The subassembly corresponding to the icosahedral point asymmetric unit (one fifth of the pentasymmetron plus one third of the trisymmetron) is outlined in gray.

The five point symmetries that can be adopted by biological assemblies are circular, dihedral, tetrahedral, octahedral and icosahedral, corresponding to Schöenflies symbols *C*, *D*, *T*, *O* and *I*, respectively. For structures with circular or dihedral symmetry, a circular symmetry parameter is required to define the number of repeats around the major symmetry axis. Examples include a viral toxin with *C*38 symmetry (Fig. 2 ▶ *c*), a clathrin cage with *D*6 symmetry (Fig. 2 ▶ *d*) and a four-layer ring with *D*17 symmetry (Fig. 2 ▶ *e*).

**Figure 3:**
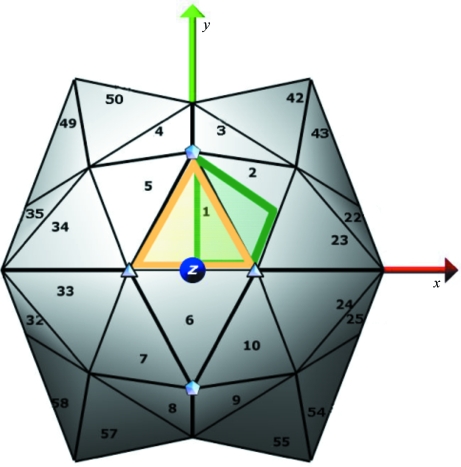
Icosahedral standard frame, shown with respect to orthogonal coordinate axes. Fivefolds and threefolds nearest to the the *z* axis are identified with symbols. Numbers show the order of symmetry operations for positions visible in this view. Yellow and green lines delimit the two alternate restricted placement boundaries for the first point asymmetric unit position.

Standard frames and hierarchical order of symmetry operations for the point symmetries are defined in Table 2 ▶. In every case the symmetry center is at the origin and symmetry elements are aligned to major orthogonal coordinate axes. The icosahedral standard frame is identical to the VIPERdb frame, with twofolds aligned to the *x*, *y*, *z* axes and fivefolds closest to the *z* axis lying in the *yz* plane (Fig. 3 ▶). Icosahedral point-symmetry operations are initiated by the application of fivefold symmetry around the vector (0, 1, ϕ), followed by application of tetrahedral symmetry operations. Where possible, the hierarchical order of symmetry operations follows the related space group: *P*23 for tetrahedral symmetry, *P*432 for octagonal symmetry.

The VIPER database restricts the position of the primary icosahedral asymmetric unit center of mass within the icosahedral standard frame (Natarajan *et al.*, 2005 ▶; Shepherd *et al.*, 2006 ▶). The advantage of restricted placement is that the transformation from an arbitrary deposited frame into the standard frame {[*P*] in (1)} has one unique solution. We utilize the same boundaries, as illustrated in Fig. 3 ▶: for triangle-shaped icosahedral asymmetric units (*e.g.* Fig. 2 ▶ *a*) the center of mass must fall within the yellow outline, or for rhomboid-shaped icosahedral asymmetric units (*e.g.* Fig. 2 ▶ *b*) within the green outline. Restricted placement conditions for the primary asymmetric unit center of mass are also defined for the other point symmetries (last row in Table 2 ▶).

#### 3.1.2. Helical symmetry

Symmetry parameters, standard frames, hierarchy of symmetry operations and asymmetric unit placement for polar and nonpolar helical symmetries are defined in Table 3 ▶. Polar and nonpolar helical symmetries closely follow the definitions for related circular and dihedral point symmetries.

Helical screw symmetry is defined using three parameters in order to allow an exact repeat: rotation around the helical axis for *n* subunit repeats, translation along the helical axis for *n* subunit repeats and number of subunit repeats divisor (*n*). For example, the fiber-diffraction structure of cucumber green mottle mosaic virus (CGMMV; Fig. 2 ▶ *f*) with 49 subunits in three turns has a rotation per subunit repeat of 1080/49 degrees and translation per subunit repeat of 70.8/49 Å. When there is no exact repeat, rotation and translation is defined for a single subunit repeat with the divisor set to unity.

Two additional parameters define rotational symmetries of a helical assembly. The presence or absence of dyad symmetry perpendicular to the helical axis distinguishes nonpolar helical structures (two ends equivalent) and polar helical structures (each end unique). Circular symmetry is a positive integer that defines the number of subunit strands twisting in parallel about the helical axis. Circular symmetry is onefold for CGMMV (Fig. 2 ▶ *f*) and fivefold for the filamentous phage illustrated in Fig. 2 ▶(*g*). Both of these helical viruses are polar.

Although not an essential parameter, the number of symmetry operations needed to generate a representative helical assembly should be defined. The number is arbitrary but should be large enough to represent the overall symmetry and all unique intersubunit interactions. It should also ideally be a multiple of the circular symmetry parameter, a multiple of 2 if dyad symmetry is present and a multiple of an odd number so that generated operations may be centered about the identity operation.

### 3.2. Transformation operations list

All transformation operations that may be applied to the deposited orthogonal angstrom coordinate positions are gathered into a single unified list. The list can include transformations to other orthogonal coordinate frames, as well as regular point, helical and crystal symmetry operations in the deposited frame. Inverse transformations (*i.e.* transformations from other frames/positions into the deposited frame/position) are not included, since they do not meet the criteria of being applicable to the deposited coordinates.

Each operation is identified by a unique ID and is represented as nine-element rotation matrix plus a three-element translation vector. To convert to the more convenient 16-­element 4 × 4 matrix form, the rotation matrix is placed in the first three rows and columns and the translation vector becomes the first three elements of the fourth column. The fourth row is set to 0, 0, 0, 1. The resulting 4 × 4 matrix that operates on four-element vectors is 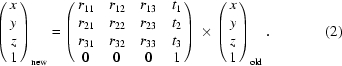

            

#### 3.2.1. Frame transformations

Assemblies in experimental orthogonal coordinate frames other than the deposited frame may be defined. The deposited frame can be any arbitrary orthogonal coordinate frame favored by the deposition authors, although a standard frame is preferred. The relationship between the deposited frame and standard point, helical, crystal and/or other frames is then explicitly defined by including frame transformations in the operations list.

#### 3.2.2. Regular symmetry operations

Point, helical or crystal symmetry operations in the deposited frame of the entry may be included in the transformation list. By convention, point-symmetry operations begin with the identity operation and the order of subsequent operations follows the hierarchy for the defined symmetry in the standard frame (*e.g.* fivefold, twofold, twofold, threefold for icosahedral symmetry; see Table 2 ▶). For point symmetries deposited in nonstandard frames, symmetry operations are calculated using (1) after determination of the frame transformation matrix [*P*] (see §[Sec sec3.1.1]3.1.1). This method ensures that relative spatial relationships among symmetry-related asymmetric units are consistent across the database. For example, the pentamer subassembly of every remediated icosahedral virus entry may be built by applying the first five point-symmetry operations. Helical symmetry operations are defined in a continuous run centered about the identity operation.

### 3.3. Assembly generation

**Figure 4:**
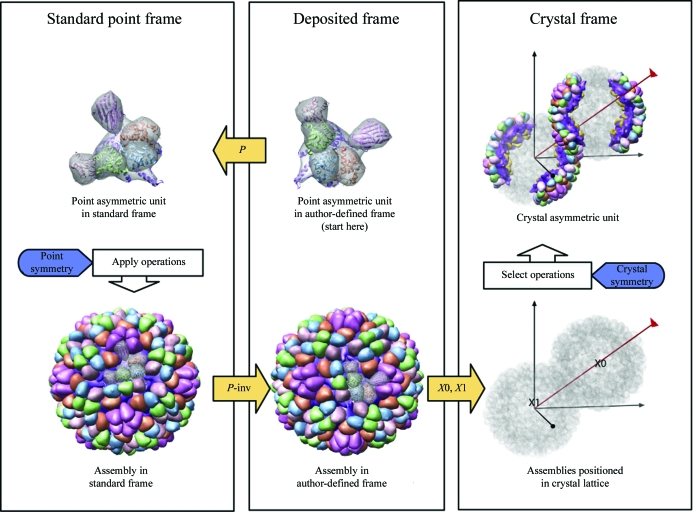
Assembly generation with regular point-symmetry example: 1al0, crystal structure of ϕX174 procapsid (Dokland *et al.*, 1997 ▶). The pathway to generate assemblies in standard point, author-defined and crystal frames is shown. Frame transformations are represented by yellow arrows connecting the deposited frame, standard icosahedral point frame and crystal frame. See §[Sec sec3.3]3.3 for details.

Here, we describe the logic for generating complete macromolecular assemblies for a PDB entry containing minimal coordinates plus a set of regular noncrystallographic symmetry operations. Fig. 4 ▶ presents an overview of generation of assemblies in multiple coordinate frames using the example of the icosahedral ϕX174 procapsid (PDB entry 1al0; Dokland *et al.*, 1997 ▶), a structure determined by X-ray crystallography with two independent virus-particle positions in the crystal asymmetric unit. Atomic coordinates were deposited in an alternate icosahedral frame.

The assembly path begins at the top center of Fig. 4 ▶ with the deposited chains represented as enveloped ribbons and pro­ceeds counterclockwise. The coordinates are moved into the standard icosahedral frame (upper left) by application of the frame-transformation matrix [*P*]. The complete biological assembly (lower left) is produced in the standard icosahedral frame by the application of 60 point-symmetry operations and is moved back to the deposited frame (bottom center) by the application of [*P*-inv], calculated as the inverse of matrix [*P*]. [*X*0] and [*X*1] are author-provided transformations that place two independent copies of the virus assembly onto the cubic (*I*2_1_3) crystal lattice body diagonal (lower right). A subset of operations defines the crystal asymmetric unit (upper right).

Assembly definitions corresponding to the path in Fig. 4 ▶ are summarized in Table 4 ▶. Each definition includes a text description and a list of one or more operation expressions with associated coordinate selections. Operation expressions are given in a compact notation and specify matrices from the operations list, which includes frame transformations [*P*], [*X*0], [*X*1] and 60 icosahedral symmetry operations, labelled 1–60, calculated in the deposited frame, [*P* ^−1^][*I*_*m*_][*P*]. An operation expression can be a comma-separated list (‘1, 5, 9’), a dash-delimited range (‘1-60’) or a matrix multiplication involving two or more lists or ranges. For instance, ‘(X0)(1-­20)’ specifies the portion of the ϕX174 procapsid crystal asymmetric unit belonging to the first independent virus particle and corresponds to the 20 transformations [*X*0][1], [*X*0][2], …, [*X*0][20]. Similarly, ‘(X1)(1-20)’ specifies the portion of the crystal asymmetric unit belonging to the second independent virus particle. The two specifications listed together define the full crystal asymmetric unit (see bottom row of Table 4 ▶). Coordinate selections are given as lists of comma-separated coordinate-group identities (Bourne *et al.*, 1997 ▶).

Complex cases such as the pseudocrystalline symmetry in icosahedral PBCV-1 (PDB entry 1m4x; Nandhagopal *et al.*, 2002 ▶) can also be represented (Figs. 2 ▶ *h* and 2 ▶ *i* and Table 5 ▶). Three deposited chains represent 1/28th of the icosahedral point asymmetric unit (yellow trimer in Fig. 2 ▶ *i*). The operations list contains 60 point-symmetry operations (‘1-60’) and 28 operations to build the icosahedral point asymmetric unit (‘61-88’). The complete capsid (Fig. 2 ▶ *h*) is built with 1680 operations specified by ‘(1-60)(61-88)’ applied to the three deposited chains. The pentasymmetron and trisymmetron sub­assemblies of PBCV-1 described by Nandhagopal and coworkers are also readily defined *via* matrix selections (Fig. 2 ▶ *i*, Table 5 ▶).

## 4. Discussion

Remediated entries for the viruses and other assemblies listed in Table 1 ▶ were released into the PDB archive on 31 July 2007 and are available by ftp or web interface from any of the wwPDB partners (RCSB PDB, EBI MSD, PDBj; see http://wwpdb.org and Berman *et al.*, 2003 ▶). PDB-format files automatically generated from remediated mmCIFs hold much of the updated information, including corrected BIOMT matrices to build the full biological assembly and a text description of the regular symmetry. For crystal structures deposited in the crystal frame, noncrystallographic symmetry operations to build the crystal asymmetric unit are provided in MTRIX records. The mmCIF files or their PDBML translations should be consulted for the most complete machine-readable representations of these entries.

One immediate consequence of remediation is that routine visualization of complete biological assemblies of viruses is now possible. Biological unit files containing explicit coordinates for the full assembly are available in the PDB archive and can be viewed with a number of different software programs. However, the downloading, storage and manipulation of a biological unit file is inefficient compared with handling the equivalent representation in matrices and co­ordinates. PBCV-1 virus (PDB entry 1m4x) is the most extreme case: the compressed storage size for the biounit file with 5040 chains is 1000 times bigger than the mmCIF or PDB file with three chains and matrices (0.3 Gb *versus* 0.3 Mb). The *Chimera Multiscale Module* was designed specifically for displaying large assemblies and can calculate full assemblies on the fly from PDB BIOMT records (Goddard *et al.*, 2005 ▶); examples of its use are shown in Figs. 2 ▶ and 4 ▶. Adoption of this mmCIF (or equivalently, PDBML) representation will further enhance the capabilities of visualization tools to display complex biological assemblies.

To optimally represent future entries of this type, we encourage the deposition of coordinates representing the minimal unique repeating unit along with a clear description of the symmetry, including all local, point, helical, two-dimensional and/or three-dimensional crystal parameters. A complete set of point-symmetry operations or representative set of helical operations should be provided in the deposited frame, along with known transformations to other experimental frames. We anticipate that continued progress in development of X-ray diffraction, cryoEM and other structural biology methods will result in many more examples of large biological assemblies with regular symmetry in years to come.

## Supplementary Material

Supplementary material file. DOI: 10.1107/S0907444908017393/mv5020sup1.pdf

## Figures and Tables

**Table 1:** Remediated entries

Symmetry type	Entry IDs, sorted by experiment type†
Circular	CryoEM: 1tja **2bk1 2bk2**
Dihedral	X-ray diffraction: **1ei7 2gtl**
	CryoEM: **1xi4 1xi5**
Icosahedral	X-ray diffraction: 1a34 1a6c 1al0 1al2 1aq3 1aq4 1ar6 1ar7 1ar8 1ar9 1asj 1auy 1aym 1ayn 1b35 1bbt 1bev 1bms 1bmv 1c8d 1c8e 1c8f 1c8g 1c8h 1c8m 1c8n 1cd3 1cov 1cwp 1d4m 1ddl 1dnv 1dwn 1dzl 1e57 1eah 1ej6 1ev1 1f15 1f2n 1f8v 1fmd 1fod 1fpn 1fpv 1fr5 1frs 1gff 1h8t 1hri 1hxs 1ihm 1hrv 1ijs 1js9 1k3v 1k5m 1laj 1lp3 1m06 1m1c 1mec 1mqt 1mst 1mva 1mvb 1mvm 1na1 1ncq 1ncr 1nd2 1nd3 1ng0 1nov 1ny7 1ohf 1ohg 1oop 1opo 1p5w 1p5y 1pgl 1pgw 1piv 1po1 1po2 1pov 1pvc 1qbe 1qgt 1qju 1qjx 1qjy 1qjz 1qqp 1r08 1r09 1r1a 1rb8 1rhi 1rmu 1ruc 1rud 1rue 1ruf 1rug 1ruh 1rui 1ruj 1rvf 1s58 1sid 1sie 1smv 1stm 1sva 1tme 1tmf 1tnv 1u1y 1uf2 1v9u 1vak 1vb2 1vb4 1vba 1vbb 1vbc 1vbd 1vbe **1vcr** 1vrh 1w39 1w8x 1wcd 1wce 1x33 1x35 1x36 1x9p 1x9t 1z14 1z1c 1z7s 1za7 1zba 1zbe 1zdh 1zdi 1zdj 1zdk 1zse 2b2d 2b2e 2b2g 2bbv 2bfu 2bny 2bpa 2bq5 2bs0 2bs1 2btv 2bu1 2buk 2c4q 2c4y 2c4z 2c50 2c51 2cas 2frp 2fs3 2fsy 2ft1 2fz1 2fz2 2g33 2g34 2g8g 2gh8 2gp1 2hwb 2hwc 2hwd 2hwe 2hwf 2iz8 2iz9 2izm 2izn 2mev 2ms2 2plv 2r04 2r06 2r07 2rm2 2rmu 2rr1 2rs1 2rs3 2rs5 2tbv 4dpv 4rhv 4sbv 5msf 6msf 7msf
	CryoEM: 1d3e 1d3i 1dgi 1dyl 1gw7 1gw8 1hb5 1hb7 1hb9 1if0 1jew 1k4r 1kvp 1ld4 1m0f 1m11 1m4x 1n6g 1na4 1nn8 1p58 1qgc 1tge 1thd 1upn 1xyr 1yxn 1z7z 1z8y 2b6b 2bld 2bvi 2c8i 2c9f 2c9g 2cse 2fte 2of6
Helical	Fiber diffraction: 1cgm 1hgv 1hgz 1hh0 1ifd 1ifi 1ifj 1ifk 1ifl 1ifm 1ifn 1ifp 1pfi 1ql1 1ql2 1rmv 1vtm 2c0w 2ifm 2ifn 2ifo 2tmv 3ifm 4ifm
	Solid state NMR: 2cox

† IDs shown in bold correspond to nonvirus structure entries.

**Table 2:** Point-symmetry representation

Point-symmetry type	Circular	Dihedral	Tetrahedral†	Octahedral†	Icosahedral‡
Schoenflies symbol	*C*	*D*	*T*	*O*	*I*
Circular symmetry	Integer *n* ≥ 1	Integer *n* ≥ 2	—	—	—
No. of operations	*n*	2*n*	12	24	60
Standard frame definition	*n*-fold on *z*	*n*-fold on *z*	Twofolds on *x*, *y*, *z*	Fourfolds on *x*, *y*, *z*	Twofolds on *x*, *y*, *z*
		Twofold on *x*	Threefolds on body diagonals	Threefolds on body diagonals	Threefolds on body diagonals
				Twofolds on plane diagonals	Fivefold vertices closest to *z* axis in *yz* plane
Hierarchy of symmetry operations	*n*-fold on *z*	*n*-fold on *z*	Twofold on *z*	Twofold on *z*	Fivefold on (0, 1, ϕ)
		Twofold on *x*	Twofold on *y*	Twofold on *y*	Twofold on *z*
			Threefold on (1, 1, 1)	Threefold on (1, 1, 1)	Twofold on *y*
				Twofold on (1, 1, 0)	Threefold on (1, 1, 1)
Asymmetric unit center-of-mass position	On +*x*	Nearest +*x* and +*z*	Between +*x*, +*z* and (1, 1, 1)	Nearest +*x* and (1, 1, 1)	*T* = 3, nearest (0, 1, ϕ) and +*z*; else nearest (0, 1, ϕ) and threefold on (ϕ/3, 0, 2ϕ + 1/3)

† Tetrahedral and octahedral standard frames and hierarchy of symmetry operations follow *International Tables for Crystallography* definitions for cubic space groups *P*23 (No. 195) and *P*432 (No. 207), respectively (Hahn, 2002 ▶).

‡ The icosahedral standard frame is identical to that utilized by VIPERdb (Reddy *et al.*, 2001 ▶), but the hierarchy of symmetry operations follows tetrahedral symmetry after the application of fivefold symmetry. ϕ = [(5)^1/2^ + 1]/2.

**Table 3:** Helical symmetry representation

Helical symmetry type	Polar	Nonpolar
No. of subunit repeats in screw definition	Integer *N* ≥ 1	Integer *N* ≥ 1
Rotation per *N* subunits around helical axis (°)	*R* × *N*, −180 < *R* ≤ 180	*R* × *N*, −180 < *R* ≤ 180
Translation per *N* subunits along helical axis (Å)	*T* × *N* > 0	*T* × *N* > 0
Dyad symmetry	No	Yes
Circular symmetry	Integer *n* ≥ 1	Integer *n* ≥ 1
No. of operations	*n* × arbitrary odd integer	2*n* × arbitrary odd integer
Standard frame definition	*n*-fold and screw on *z*	*n*-fold and screw on *z*
		Twofold on *x*
Hierarchy of symmetry operations	*n*-fold on *z*	*n*-fold on *z*
	Screw on *z*	Twofold on *x*
		Screw on *z*
Asymmetric unit center-of-mass position	On +*x*	Nearest +*x* and +*z*

**Table 4:** Assembly definitions, icosahedral virus crystal illustrated in Fig. 4 ▶

Assembly description	Frame	Operation expression	Coordinate groups
Complete assembly	Deposited	1-60	*A*, *B*, *C*, *D*, *E*, *F*, *G*
Pentamer	Deposited	1-5	*A*, *B*, *C*, *D*, *E*, *F*, *G*
Complete assembly	Icosahedral	(P)(1-60)	*A*, *B*, *C*, *D*, *E*, *F*, *G*
Point asymmetric unit	Icosahedral	(P)(1)	*A*, *B*, *C*, *D*, *E*, *F*, *G*
Crystal asymmetric unit	Crystal	(X0)(1-20)	*A*, *B*, *C*, *D*, *E*, *F*, *G*
		(X1)(1-20)	*A*, *B*, *C*, *D*, *E*, *F*, *G*

**Table 5:** Assembly definitions, complex symmetry (PBCV-1)

Assembly description	Frame	Operation expression	Coordinate groups
Complete assembly	Deposited	(1-60)(61-88)	*A*, *B*, *C*
Point asymmetric unit	Deposited	61-88	*A*, *B*, *C*
Trisymmetron	Deposited	(1, 10, 23)(61, 68-88)	*A*, *B*, *C*
Pentasymmetron	Deposited	(1-5)(62-67)	*A*, *B*, *C*

## Footnotes

1 Supplementary material has been deposited in the IUCr electronic archive (Reference: MV5020). Services for accessing this material are described at the back of the journal.
