# Supplementary material for: Representation of viruses in the remediated PDB archive
Source: Acta Crystallogr D Biol Crystallogr. 2008 Jul 17;64(Pt 8):874–82. doi: 10.1107/S0907444908017393 (PMC2677383; doi:10.1107/S0907444908017393)
Supplement: Supplementary file 1 [file d-64-00874-sup1.pdf]

```
#  SUPPLEMENT 1 FOR
# Representation of Viruses in the Remediated PDB Archive
# C. L. Lawson, K. Henrick, J. Westbrook, S. Dutta, & H. M. Berman
#
# Below is an abbreviated mmcif representation for PDB entry 1AL0, a virus
# structure used as an example in the manuscript.
#
# data item definitions can be found at http://mmcif.pdb.org
#
data_1AL0
#
_entry.id   1AL0
#
_cell.entry_id           1AL0
_cell.length_a           774.000
_cell.length_b           774.000
_cell.length_c           774.000
_cell.angle_alpha        90.00
_cell.angle_beta         90.00
_cell.angle_gamma        90.00
#
_symmetry.entry_id                          1AL0
_symmetry.space_group_name_H-M              'I 21 3'
#
loop_
_entity.id
_entity.type
_entity.src_method
_entity.pdbx_description
_entity.formula_weight
_entity.pdbx_number_of_molecules
_entity.details
1 polymer nat 'SCAFFOLDING PROTEIN GPD' 16953 4 ?
2 polymer nat 'CAPSID PROTEIN GPF'      48423 1 ?
3 polymer nat 'SPIKE PROTEIN GPG'       19061 1 ?
4 polymer nat 'SCAFFOLDING PROTEIN GPB' 13863 1 ?
#
loop_
_struct_asym.id
_struct_asym.pdbx_blank_PDB_chainid_flag
_struct_asym.pdbx_modified
_struct_asym.entity_id
_struct_asym.details
A N N 1 ?
B N N 1 ?
C N N 1 ?
D N N 1 ?
E N N 2 ?
F N N 3 ?
G N N 4 ?
#
_pdbx_point_symmetry.entry_id              1AL0
_pdbx_point_symmetry.Schoenflies_symbol    I
#
loop_
_pdbx_struct_oper_list.id
_pdbx_struct_oper_list.type
_pdbx_struct_oper_list.matrix[1][1]
_pdbx_struct_oper_list.matrix[1][2]
_pdbx_struct_oper_list.matrix[1][3]
_pdbx_struct_oper_list.vector[1]
_pdbx_struct_oper_list.matrix[2][1]
_pdbx_struct_oper_list.matrix[2][2]
_pdbx_struct_oper_list.matrix[2][3]
_pdbx_struct_oper_list.vector[2]
_pdbx_struct_oper_list.matrix[3][1]
_pdbx_struct_oper_list.matrix[3][2]
_pdbx_struct_oper_list.matrix[3][3]
_pdbx_struct_oper_list.vector[3]
```

```
P  'transform to point frame'
0.00000000  -1.00000000 0.00000000  0.00000
1.00000000  0.00000000  0.00000000  0.00000
0.00000000  0.00000000  1.00000000  0.00000
X0 'transform to crystal frame'
1.00000000  0.00000000  0.00000000  188.08200
0.00000000  1.00000000  0.00000000  188.08200
0.00000000  0.00000000  1.00000000  188.08200
X1 'transform to crystal frame'
0.83425300  0.46385000  -0.29810300 -4.02480
-0.29810300 0.83425300  0.46385000  -4.02480
0.46385000  -0.29810300 0.83425300  -4.02480
1  'point symmetry operation'
1.00000000  0.00000000  0.00000000  0.00000
0.00000000  1.00000000  0.00000000  0.00000
0.00000000  0.00000000  1.00000000  0.00000
2  'point symmetry operation'
0.50000000  -0.80901699 0.30901699  0.00000
0.80901699  0.30901699  -0.50000000 0.00000
0.30901699  0.50000000  0.80901699  0.00000
3  'point symmetry operation'
-0.30901699 -0.50000000 0.80901699  0.00000
0.50000000  -0.80901699 -0.30901699 0.00000
0.80901699  0.30901699  0.50000000  0.00000
#
# (point symmetry operations 4-59 omitted for brevity)
#
60 'point symmetry operation'
-0.30901699 0.50000000  -0.80901699 0.00000
0.50000000  0.80901699  0.30901699  0.00000
0.80901699  -0.30901699 -0.50000000 0.00000
#
loop_
_pdbx_struct_assembly.id
_pdbx_struct_assembly.details
1   'complete icosahedral assembly'
2   'icosahedral asymmetric unit'
3   'icosahedral pentamer'
4   'icosahedral 23 hexamer'
PAU 'icosahedral asymmetric unit, std point frame'
XAU 'crystal asymmetric unit, crystal frame'
#
loop_
_pdbx_struct_assembly_gen.assembly_id
_pdbx_struct_assembly_gen.oper_expression
_pdbx_struct_assembly_gen.asym_id_list
1   (1-60)           A,B,C,D,E,F,G
2   1                A,B,C,D,E,F,G
3   (1-5)            A,B,C,D,E,F,G
4   (1,2,6,10,23,24) A,B,C,D,E,F,G
PAU P                A,B,C,D,E,F,G
XAU (X0)(1-20)       A,B,C,D,E,F,G
XAU (X1)(1-20)       A,B,C,D,E,F,G
```
